# Factors Determining Home versus Rehabilitation Discharge Following Primary Total Joint Arthroplasty for Patients Who Live Alone

**DOI:** 10.3390/geriatrics5010007

**Published:** 2020-02-12

**Authors:** Christopher Fang, Sara J. Lim, David J. Tybor, Joseph Martin, Mary E. Pevear, Eric L. Smith

**Affiliations:** 1 Boston University School of Medicine, Boston, MA 02118, USA; cfang@bu.edu (C.F.); sarajlim@bu.edu (S.J.L.); 2 Department of Public Health and Community Medicine, Tufts University School of Medicine, Boston, MA 02111, USA; david.tybor@tufts.edu; 3 Vanderbilt University, Nashville, TN 02118, USA; joseph.t.martin@vanderbilt.edu; 4 Department of Orthopedic Surgery, Boston Medical Center, 725 Albany St, Boston, MA 02118, USA; mary.pevear@gmail.com; 5 New England Baptist Hospital, 125 Parker Hill Ave, Boston, MA 02120, USA

**Keywords:** TJA, TKA, THA, Discharge Status, Skilled Nursing Facility

## Abstract

Patients who are discharged home following primary hip and knee arthroplasty have lower associated costs and better outcomes than patients who are discharged to skilled nursing facilities (SNFs). However, patients who live alone are more likely to be discharged to an SNF. We studied the factors that determine the discharge destination for patients who live alone after total joint arthroplasty (TJA) at an urban tertiary care academic hospital between April 2016 and April 2017. We identified 127 patients who lived alone: 79 (62.2%) were sent home, and 48 (37.8%) were sent to an SNF after surgery. Patients who went home versus to an SNF differed in age, employment status, exercise/active status, patient expectation of discharge to an SNF, ASA score, and the length of stay. After controlling for expectations of discharge to an SNF (OR: 28.98), patients who were younger (OR: 0.03) and employed (OR: 6.91) were more likely to be discharged home. In conclusion, the expectation of discharge location was the strongest predictor of discharge to an SNF even after controlling for age and employment. Future research should include a multi-hospital approach to strengthen the validity of our findings and investigate additional factors that impact discharge destination.

## 1. Introduction

In the United States, total joint arthroplasty (TJA) demand has increased due to the aging population and rising obesity rates [1]. TJA is also the most effective treatment for advanced arthritis of the hip and knee [2,3]. Concurrent with the increase in TJAs, health care reform has continued to place emphasis on cost-effective care, including the Centers for Medicare and Medicaid Services advocating for a reduction in TJA costs [4]. A major contributor to the cost is the post-operative discharge destination to skilled nursing facilities (SNF), which accounted for 49% of all patients and 70% of post-discharge costs in 2014 [5].

Patients discharged to home following TJA have lower associated costs, decreased rates of infection, increased patient satisfaction, reduced 30-day readmissions, and improved clinical outcomes [6,7,8,9,10,11]. Factors driving discharge destination include age, race/ethnicity, socioeconomic status (SES), and access to health care [12,13,14,15,16,17,18,19,20,21]. Living status has been shown to be a significant factor in discharge destination, with patients living alone being more likely to be discharged to an SNF [12]. However, it is unknown which factors contribute to discharge destination for this specific population. With the transition to bundled care payments, there has been growing pressure to reduce costs that are associated with healthcare, and the ability to predict discharge destination is becoming increasingly important to accomplishing this task. The purpose of this observational study was to determine factors associated with patients who, despite living alone, are discharged to home.

## 2. Materials and Methods

### 2.1. Data Collection

After institutional review board approval, a single-center cohort study was conducted with 615 consecutive patients who underwent a primary TJA at our tertiary care academic medical center between April 2016 and April 2017. Patients were identified by using Current Procedural Terminology codes 27130 (total hip arthroplasty) and 27447 (total knee arthroplasty). We reviewed the charts and excluded patients who did not live alone, who underwent revision TJA, who were discharged to hospice, who left against medical advice, or who had an unknown discharge destination. These patients were then separated into two groups depending on their discharge destination: home (with or without home rehabilitation services, *n* = 79) versus a skilled nursing facility (SNF, *n* = 48).

Data were collected prospectively for each patient included in the study. Age, gender, pre-operative weight, body mass index (BMI), American Society of Anesthesiologists (ASA) score [22], diabetes status, smoking status, intravenous (IV) drug use, depression, primary language, surgery type (total knee arthroplasty or total hip arthroplasty), previous surgery, race/ethnicity, insurance and employment status were extracted from the pre-operative anesthesia note; all were pre-operatively self-reported by the patient with the exception of BMI, which was measured by nursing in the clinic. To assess health status, we used ASA score rather than quantifying number of comorbidities due to the uncertainty of the impact of each comorbidity on health. Exercise/active status was determined by two researchers who independently adjudicating mobility without the aid of devices other than a cane from the pre-operative notes. Proximity to hospital was determined by geocoding with the use of the patient’s home address. We assessed patient SES by using estimated median household income in 2015 by zip code as reported by a city-data census [23], and these data were subdivided into 3 categories: low, middle and high, as previously published [17]. We examined the pre-operative discharge planner’s notes for explicit patient statements of preference for SNF discharge. The length of stay in hospital was defined as days post-surgery spent in the hospital. The post-operative pain level was collected from physical and occupational therapy notes at initial assessment, and it was recorded by the visual analog scale with a range of 0–10.

### 2.2. Statistical Analysis

We used independent samples t-tests, Fisher’s exact test, and the Mann–Whitney U tests to compare continuous and categorical variables, as appropriate. We used binary logistic regression models to identify significant univariate predictors of whether a patient was discharged to home. Age (log-transformed), employment, and expectation of discharge to an SNF were included in the multiple logistic regression analysis. All analyses were conducted by using STATA v14 (StataCorp, College Station, TX, USA) [24] with a two-sided alpha level of 0.05.

## 3. Results

### 3.1. Univariate Analysis

One hundred and twenty-seven patients met the inclusion criteria, with 79 (62.2%) discharged to home. There was a significant difference in age (*p* = 0.004), employment (*p* = 0.005), exercise/active status (*p* = 0.03), expectation of discharge to an SNF (*p* ≤ 0.0001), ASA score (*p* = 0.0003), and length of stay in the hospital (*p* = 0.013) between patients living alone who were discharged to home versus SNFs (Table 1). There were no significant differences in gender, BMI, smoking, IV drug use, depression, previous surgery, SES, race/ethnicity, proximity to hospital, insurance type, language, diabetes, surgery type and pain level. Patients living alone were more likely to be discharged to home if they were younger, employed, active, in the hospital for a shorter amount of time, had no expectation of being discharged to an SNF, and had a lower ASA score.

### 3.2. Multivariate Analysis

In a multiple logistic regression model, age, employment, and the expectation of being discharged to an SNF were significant predictors of home discharge (Table 2)**.** Patient preference was the strongest predictor of discharge destination, with those who wanted to attend an SNF being 29 times more likely to do so (odds ratio (OR): 28.98, 95% confidence interval (CI): 8.87–94.67). Younger patients were found to be more likely to go home (OR: 0.033, 95% CI: 0.001–0.831), and employed patients were found to be more likely to go home relative to patients who were not currently employed (OR: 6.91, 95% CI: 1.02–46.92) (Table 2). All other univariate factors, including ASA, exercise/active status and length of stay, were not significant after controlling for expectations of rehabilitation discharge.

## 4. Discussion

A discharge to an SNF after TJA is associated with poorer outcomes, higher rates of infection, and increased costs [6,7,8,9,10,11]. Identifying factors that influence discharge destination might have a positive impact on post-operative outcomes. Our study confirmed that even in patients who live alone, patient expectation is the most important predictor of discharge destination [15].

In addition, we found that younger patients and employed patients were more likely to be discharged to home after controlling for discharge expectation. These findings reflect that younger patients may be healthier and not require the services provided at an SNF. In addition, those who are employed may desire to return quickly to work and would therefore would not prefer discharge to an SNF. Future research is needed to explore the SES factors for employment.

We identified several patients who requested discharge to an SNF due to a previous positive experience after a different type of surgery or a fear that being alone would lead to a poorer health outcome. Better pre-operative patient education on the merits of home discharge, even for patients who live alone, might increase the number of patients being sent home and thereby improve post-operative outcomes. Additionally, our study identified that many patients who were discharged home were able to recruit nearby family or friends for additional social support post-operatively. A social support component could be added to existing pre-operative patient education programs to emphasize that having enough support coupled with home discharge ultimately results in fewer complications, better outcomes, and lower expenses.

Public versus private insurance was not a significant predictor of discharge destination. This may indicate a changing healthcare landscape with less health inequities when it comes to the cost barrier that is associated with employing a visiting nurse association service after discharge to home. IV drug use and race/ethnicity were not statistically different, but this may be a type II error due to the small sample size. Further research is needed.

Previous studies have shown that patients who live alone are disproportionately sent to SNFs [12]. Similar studies on all patient populations have shown similar factors such as age, ethnicity, socioeconomic status, and access to health care [12,13,14,15,16,17,18,19,20,21], but our study is the first to directly identify factors influencing discharge to home specifically for this patient population following a primary TJA. These results reveal possible paths of patient education and social support to ameliorate their post-operative outcome. Additionally, the unique nature of the large urban tertiary care academic hospital setting that employs an up-to-date patient education program on discharge allowed for a diverse study pool that revealed clear factors that kept patients from receiving home destination.

The major limitations of this study are the reliance on self-reported data and the small sample size. A larger sample size would result in more precise estimates and reduce the likelihood of type II error for predictors such as race/ethnicity and IV drug use. Further research in different populations is needed to determine variations due to geographic location or demographics. In addition, the role of SES must be investigated to see potential correlations.

In conclusion, we found that no expectation of discharge to an SNF, younger age, and positive employment status were significant indicators for discharge to home in patients who live alone following primary total knee or total hip arthroplasty. Our findings suggest that pre-operative patient education that emphasizes the benefits of home discharge, thereby minimizing expectation of a discharge to an SNF, could result in a greater frequency of home discharge. Resource allocation can be driven towards pre-operatively questioning expectations for discharge for patients who are identified as living alone. Clinically, this could result in better post-operative outcomes and decreased costs, as well as better predictability for bundled care payments.

## Figures and Tables

**Table 1:** Factors for patients living alone discharged to home or skilled nursing facility following TJA.

Factor	Home (*n* = 79)	SNF Group (*n* = 48)	*p*-Value
Total, n (%)	79 (62.2%)	48 (37.8%)	
Age (+/−SD)	61.64 +/− 10.67	67.10 +/− 9.43	*p* = 0.0042 *
Gender, male, (%)	28 (35%)	11 (23%)	*p* = 0.167
BMI (+/− SD, range)	32.18 +/− 6.78 range = 44.3	33.85 +/− 5.88 range = 24.4	*p* = 0.1617
Currently smoking, yes, (%)	22 (27.85%)	10 (20.8%)	*p* = 0.408
IV drug use (%)	9 (11.39%)	1 (2.08%)	*p* = 0.088
Depression	35 (44.30%)	18 (37.50%)	*p* = 0.465
Previous surgery	74 (93.67%)	45 (93.75%)	*p* = 0.999
Employed			*p* = 0.005 *
Unemployed/Retired	52 (65.82%)	39 (81.25%)	
Employed	21 (26.58%)	2 (4.17%)	
Unknown	6 (7.59%)	7 (14.58%)	
SES (based on Zip Code)			*p* = 0.828
High	13 (16.46%)	7 (14.58%)	
Middle	40 (50.63%)	27 (56.25%)	
Low	26 (32.91%)	14 (29.17%)	
Race/Ethnicity			*p* = 0.065
Unknown	26 (32.90%)	23 (47.92%)	
White	17 (21.52%)	11 (22.92%)	
Hispanic	16 (20.25%)	2 (4.17%)	
Black	20 (25.32%)	12 (25%)	
Proximity to hospital (miles) (+/− SD, range)	9.5 +/− 14.52 range = 87	14.54792 +/− 26.31 range = 135.7	*p* = 0.1665
Insurance Type			*p* = 0.394
Unknown	7 (8.86%)	2 (4.17%)	
Public	57 (72.15%)	33 (68.75%)	
Private	15 (18.99%)	13 (27.08%)	
Exercise/active status	53 (67.09%)	22 (45.83%)	*p* = 0.025 *
Preferred language			*p* = 0.344
English	64 (81.01%)	43 (89.58%)	
Spanish	9 (11.39%)	2 (4.17%)	
Other	6 (7.59%)	3 (6.25%)	
Diabetes Mellitus type II	16 (20.25%)	14 (29.17%)	*p* = 0.285
Expectation of discharge to SNF	6 (7.59%)	31 (64.58%)	*p* ≤ 0.001 *
ASA score (median, IQR)	2, 2–3	3, 3–3	*p* = 0.0005 *
Total Knee Arthroplasty	48 (60.76%)	25 (52.08%)	*p* = 0.36
Length of Stay in Hospital (days) (+/− SD, range)	2.7413 +/− 1.315 range = 9	3.5729 +/− 2.385 range = 11	*p* = 0.0126 *
Pain level (0–10) (median, IQR)	6, 4–8	6, 5–8	*p* = 0.3661

(*) denotes significant *p*-value.

**Table 2:** Multiple logistic regression model predicting discharge to home after TJA.

Factor	Odds Ratio	*p*-Value	95% CI
Age in years (log)	0.033	0.038	0.001—0.831
Employment			
Employed	6.914	0.048	1.019—46.920
Unknown	0.412	0.211	0.103—1.652
Unemployed	Ref.	—	—
Expectation of discharge to SNF	28.977	< 0.001	8.869—94.672

## References

[1] Wilson N.A., Schneller E.S., Montgomery K., Bozic K.J. (2008). Hip and knee implants: Current trends and policy considerations. Health Aff..

[2] Derman P.B., Fabricant P.D., David G. (2014). The Role of Overweight and Obesity in Relation to the More Rapid Growth of Total Knee Arthroplasty Volume Compared with Total Hip Arthroplasty Volume. J. Bone Jt. Surg. Am..

[3] Kurtz S., Ong K., Lau E., Mowat F., Halpern M. (2007). Projections of primary and revision hip and knee arthroplasty in the United States from 2005 to 2030. J. Bone Jt. Surg. Am..

[4] Centers for Medicare & Medicaid Services (CMS), HHS (2015). Medicare Program; Comprehensive Care for Joint Replacement Payment Model for Acute Care Hospitals Furnishing Lower Extremity Joint Replacement Services. Fed. Regist..

[5] Bozic K.J., Ward L., Vail T.P., Maze M. (2014). Bundled payments in total joint arthroplasty: Targeting opportunities for quality improvement and cost reduction. Clin. Orthop. Relat. Res..

[6] Froimson M.I. (2013). In-home care following total knee replacement. Cleve Clin. J. Med..

[7] Jorgenson E.S., Richardson D.M., Thomasson A.M., Nelson C.L., Ibrahim S.A. (2015). Race, Rehabilitation, and 30-Day Readmission After Elective Total Knee Arthroplasty. Geriatr. Orthop. Surg. Rehabil..

[8] Keswani A., Weiser M.C., Shin J., Lovy A.J., Moucha C.S. (2016). Discharge Destination After Revision Total Joint Arthroplasty: An Analysis of Postdischarge Outcomes and Placement Risk Factors. J. Arthroplast..

[9] Harada N.D., Chun A., Chiu V., Pakalniskis A. (2000). Patterns of rehabilitation utilization after hip fracture in acute hospitals and skilled nursing facilities. Med. Care.

[10] Tian W., DeJong G., Horn S.D., Putman K., Hsieh C.H., DaVanzo J.E. (2012). Efficient rehabilitation care for joint replacement patients: Skilled nursing facility or inpatient rehabilitation facility?. Med. Decis. Mak..

[11] Mallinson T.R., Bateman J., Tseng H.Y., Manheim L., Almagor O., Deutsch A., Heinemann A.W. (2011). A comparison of discharge functional status after rehabilitation in skilled nursing, home health, and medical rehabilitation settings for patients after lower-extremity joint replacement surgery. Arch. Phys. Med. Rehabil..

[12] Pablo P.D., Losina E., Phillips C.B., Fossel A.H., Mahomed N., Lingard E.A., Katz J. (2004). Determinants of discharge destination following elective total hip replacement. Arthritis Rheum.

[13] Francis M.L., Scaife S.L., Zahnd W.E., Cook E.F., Schneeweiss S. (2009). Joint replacement surgeries among medicare beneficiaries in rural compared with urban areas. Arthritis Rheum.

[14] Freburger J.K., Holmes G.M., Ku L.J.E., Cutchin M.P., Heatwole-Shank K., Edwards L.J. (2011). Disparities in post-acute rehabilitation care for joint replacement. Arthritis Care Res..

[15] Halawi M.J., Vovos T.J., Green C.L., Wellman S.S., Attarian D.E., Bolognesi M.P. (2015). Patient expectation is the most important predictor of discharge destination after primary total joint arthroplasty. J. Arthroplast..

[16] Inneh I.A. (2015). The Combined Influence of Sociodemographic, Preoperative Comorbid and Intraoperative Factors on Longer Length of Stay After Elective Primary Total Knee Arthroplasty. J. Arthroplast..

[17] Inneh I.A., Clair A.J., Slover J.D., Iorio R. (2016). Disparities in Discharge Destination After Lower Extremity Joint Arthroplasty: Analysis of 7924 Patients in an Urban Setting. J. Arthroplast..

[18] Inneh I.A., Iorio R., Slover J.D., Bosco J.A. (2015). Role of Sociodemographic, Co-morbid and Intraoperative Factors in Length of Stay Following Primary Total Hip Arthroplasty. J. Arthroplast..

[19] Morrow-Howell N., Proctor E. (1994). Discharge destinations of Medicare patients receiving discharge planning: Who goes where?. Med. Care.

[20] Munin M.C., Kwoh C.K., Glynn N., Crossett L., Rubash H.E. (1995). Predicting discharge outcome after elective hip and knee arthroplasty. Am. J. Phys. Med. Rehabil..

[21] Sharareh B., Le N.B., Hoang M.T., Schwarzkopf R. (2014). Factors determining discharge destination for patients undergoing total joint arthroplasty. J. Arthroplast..

[22] Doyle D.J., Garmon E.H. American Society of Anesthesiologists Classification (ASA Class). http://www.asahq.org/For-Members/Clinical-Information/ASA-Physical-StatusClassification-System.aspx.

[23] City-Data Census. http://www.city-data.com/.

[24] StataCorp 2015 (2015). Stata Statistical Software: Release 14.

